# Treatment outcomes of multidisciplinary management of recurrent urinary tract infections: A 2‐year experience in a dedicated complex‐UTI clinic

**DOI:** 10.1002/bco2.70177

**Published:** 2026-03-04

**Authors:** Pragnitha Chitteti, Ekpeno Inyang, Ahmed Ghonaimy, Jayne Morris‐Laverick, Stephanie Bezemer, Igor Kubelka, Victoria McCune, Mehwash Nadeem

**Affiliations:** ^1^ Department of Urology James Cook University Hospital Middlesbrough UK; ^2^ Department of Microbiology James Cook University Hospital Middlesbrough UK; ^3^ School of Medicine Newcastle University Newcastle Upon Tyne UK

**Keywords:** antimicrobial resistance, dedicated complex‐UTI clinic, Intravesical therapy, multidisciplinary approach, quality of life, recurrent urinary tract infections

## Abstract

**Objectives:**

This work aimed to assess treatment outcomes in patients with recurrent urinary tract infections (rUTIs) managed in a dedicated multidisciplinary complex‐UTI clinic. In addition, this work aimed to evaluate the impact of tailored interventions on patient‐reported outcomes, including quality of life (QoL) and symptomatic improvement.

**Materials and Methods:**

A 2‐year, single‐centre, prospective observational cohort study was conducted in a dedicated tertiary care UTI clinic. A multidisciplinary team comprising urologists, microbiologists and specialist nurses developed individualized management plans based on thorough assessments, including patient history, physical examination and necessary investigations. Data variables included patient demographics, urine culture results with antibiotic sensitivities, investigations performed and the treatment options administered. The minimum follow‐up period was 6 months after the implementation of treatment in the clinic. Pretreatment and posttreatment QoL were assessed, along with posttreatment Patient Global Impression of Improvement (PGI‐I) scores.

**Results:**

A total of 211 patients (mean age: 58.3 years, 89.6% female) were included. First‐ and second‐line treatments were effective for 71% of patients, while 29% required tertiary interventions. Post‐treatment, 81% of patients reported good‐to‐excellent improvement on the PGI‐I scale, and 70% achieved good‐to‐excellent QoL. Overall treatment success, defined as reduced UTI frequency or symptom resolution, was reported in 87.7% of patients. Factors such as immunosuppression and history of hospital admission for urosepsis were associated with poor treatment outcomes with statistical significance.

**Conclusion:**

Dedicated multidisciplinary UTI clinics significantly improve treatment outcomes and QoL for patients with rUTIs. The findings support broader adoption of multidisciplinary approaches, emphasizing early intervention and tailored care to optimize clinical efficiency and enhance the patient experience.

## 1 INTRODUCTION

Urinary tract infections (UTIs) are among the most common bacterial infections globally, affecting individuals across all ages and genders. Recurrent UTIs (rUTIs), as defined by the European Association of Urology (EAU), involve three or more episodes within 1 year or two or more within 6 months, presenting substantial challenges for both patients and healthcare systems.^1^, ^2^ The financial burden of UTIs is significant, encompassing direct medical costs as well as indirect costs from absenteeism, reduced productivity and broader societal impacts. In the United States alone, UTIs lead to approximately 10.5 million medical visits annually, contributing substantially to healthcare expenditures. rUTIs further intensify this burden through repeated consultations, diagnostic tests, treatment cycles and, in severe cases, hospital admissions.^3^

This issue is compounded by the global rise in antimicrobial resistance (AMR), which complicates rUTI management. Misuse and overuse of antibiotics have fostered the emergence of resistant uropathogens, diminishing the effectiveness of standard therapies. Community‐acquired antibiotic‐resistant UTIs are associated with increased healthcare costs and greater risks of complications such as pyelonephritis and urosepsis.^4^, ^5^ Infections caused by multidrug‐resistant organisms require broader‐spectrum or more toxic antibiotics, escalating treatment costs, adverse effects and the risk of further resistance.^6^

In response, various alternative treatment strategies have gained prominence, including methenamine hippurate, cranberry products, D‐mannose, vaginal oestrogen, probiotics, urine alkalinization, immunotherapy, intravesical therapies and behavioural modifications such as increased fluid intake, postcoital voiding and proper perineal hygiene.^7^, ^8^, ^9^, ^10^

Given the rise in rUTIs that are refractory to conventional treatments, a collaborative multidisciplinary approach involving urologists, microbiologists and specialist nurses is essential. The authors believe that a well‐planned treatment regimen tailored for each patient facilitates targeted but holistic care. Additionally, the ALTAR and AnTIC studies have highlighted the poor diagnostic performance of standard midstream urine (MSU) cultures, which often yield negative results despite convincing clinical evidence of urinary infection.^11^, ^12^ This adds another layer of difficulty to treating rUTIs in the absence of an antibiogram. In such cases, the readily available input of clinical microbiologists becomes crucial to avoid unnecessary antibiotic use. Dedicated UTI clinics provide comprehensive evaluation with thorough assessments, including imaging and cystoscopic examination where indicated, to identify underlying anatomical or functional causes. These clinics also facilitate coordinated multidisciplinary care that integrates both acute treatment and long‐term prevention while enhancing patient education.^13^

Despite these advances, there is limited literature on overall outcomes associated with multidisciplinary management of rUTIs in dedicated UTI clinics. This study aims to evaluate the treatment outcomes in a complex rUTI cohort managed through a *dedicated complex‐UTI clinic with multidisciplinary input*.

## 2 MATERIALS AND METHODS

### 2.1 Study design

This is a single‐centre prospective observational cohort study conducted at a tertiary referral centre with a dedicated complex‐UTI clinic. The study was approved by the institutional audits and clinical governance team.

The primary aim was to assess treatment outcomes in patients with recurrent UTIs managed through a multidisciplinary approach involving urologists, specialist nurses and microbiologists.

### 2.2 Study setting

**FIGURE 1:**
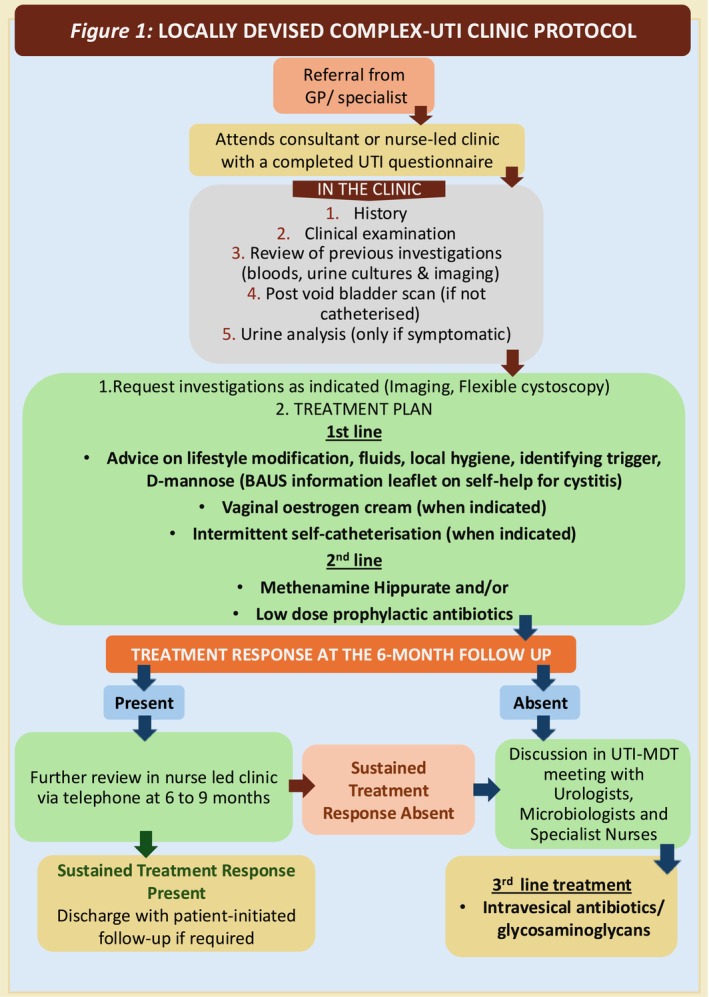
Locally devised complex‐UTI clinic protocol.

The complex‐UTI clinic was established by the Urology Department in collaboration with microbiology. The clinic's structure and treatment protocol were locally designed (Figure 1) and approved through multidisciplinary team (MDT) discussions involving functional urologists, functional urology specialist nurses and microbiologists. Final approval was granted by the clinical effectiveness committee.

Patients were referred by GPs or by secondary and tertiary specialists across various disciplines. Upon referral, patients were mailed a locally designed, trust‐approved UTI questionnaire (Appendix S1) to gather detailed clinical history and information on previous treatment, prior to attending the clinic. At their initial appointment with either a consultant or specialist nurse, patients submitted the completed questionnaire. A detailed history and physical examination were conducted, along with a review of previous investigations including blood tests, urine cultures and imaging where available. Postvoid bladder scans were performed for all patients, except those with indwelling catheters or performing intermittent self‐catheterization. Urine analysis was performed for patients exhibiting active symptoms of UTI at the time. Further investigations including repeat urine culture and sensitivity tests, flexible cystoscopy or renal tract imaging were requested where clinically indicated, following standard guidelines.^14^, ^15^

Based on the information from the questionnaire and clinic assessment, a personalized treatment plan was developed.

*First‐line treatment* included providing verbal and written advice (via British Association of Urological Surgeons [BAUS] leaflets and website) on lifestyle modification, fluid intake, hygiene and UTI trigger avoidance.^16^, ^17^ Additional first‐line interventions included topical vaginal oestrogen for perimenopausal and postmenopausal women, self‐catheterization training for those with incomplete emptying, and over‐the‐counter D‐mannose and probiotics.

*Second‐line treatment* involved prescribing methenamine hippurate with vitamin C and/or low‐dose prophylactic antibiotics. A combination of first‐ and second‐line options was employed at the first instance. Surgical interventions were planned where indicated.

Once the initial management with first‐ and second‐line treatment was started, the patients were followed up at 6 months with interim access to specialist nurse support for any help or advice over the telephone. At the 6‐month follow up, treatment response was assessed. If improvement was observed, the treatment was continued with further reviews every 6 to 9 months. Patients showing sustained benefit were eventually discharged with an open‐access, patient‐initiated follow‐up option active for 12 months after discharge to address symptom recurrence.^18^

Patients without improvement were discussed at the monthly complex‐UTI MDT meetings involving urologists, microbiologists and specialist nurses. In addition, patients with mixed growth on cultures and lack of treatment response to antibiotics despite positive cultures were also discussed with the microbiologist allowing for full speciation of mixed growth samples. The MDT focused on identifying modifiable risk factors, refining treatment strategies and ordering additional investigations where necessary (e.g., STI or Mycobacterium screening). Based on MDT consensus, an appropriate decision for further management in the form of tertiary or third‐line treatment was made.

*Third‐line treatment* included intravesical glycosaminoglycan therapy, antibiotic therapy or surgical management, where necessary. A clinical decision was made taking into consideration the patient and disease factors, culture and sensitivity patterns, along with the patient's expectation and wishes. Additionally, selected patients meeting eligibility criteria were referred to an ongoing multicentre, randomized, double‐blind, placebo‐controlled UTI vaccine trial following MDT discussion.^19^

### 2.3 Study population

For this study, we included all the patients who were referred to and seen in the out‐patient complex UTI clinic over a 2‐year period, from April 2021 to March 2023. A minimum follow‐up time of 6 months after initiation of treatment in the UTI clinic was deemed to be essential to include these patients for the analysis. All the patients during this time period were included to ensure a fixed sample size and to minimize selection bias. Patients who discontinued the advised treatment regimen before the 6‐month follow‐up and those who were lost to follow‐up were planned for exclusion from the study.

### 2.4 Data collection, definitions and study outcomes

The data were collected from a prospectively maintained database of all the patients referred to the UTI clinic. This database contained patient data collated from electronic, as well as paper‐based healthcare records. The relevant demographic and clinical data were collected. The data variables collected and analysed included patient‐related parameters like age, gender, source of referral to the clinic (GP/Specialist), medical co‐morbidities, menopausal status among female patients, frequency of UTI episodes and history of hospital admission with urosepsis. Investigation‐related data variables included the bacterial growth on urine cultures and their antibiotic sensitivity pattern along with findings on renal tract imaging and flexible cystoscopy, where performed (based on clinician's discretion). Data regarding the treatment received before referral to the UTI clinic and treatment provided at the UTI clinic were recorded. Pretreatment quality of life (QoL) was recorded by using the QoL component of the International Prostate Symptom Score and categorized into good to excellent (0–1 on IPSS‐QoL), satisfactory (2–3 on IPSS‐QoL) and poor (4–6 on IPSS‐QoL) QoL categories.^20^

*Significant comorbidity* was defined as ‘the presence of three or more comorbidities such as systemic hypertension, diabetes mellitus, stroke, autoimmune diseases and malignancies that increased frailty of the patients’. Any patient on immunosuppressive medication, chemotherapy or disease modifying agents was considered as *immunosuppressed*. *Neuropathy* referred to the presence of neurological conditions affecting the bladder such as multiple sclerosis, Parkinson's disease, stroke, spina bifida or spinal cord injuries.

*UTI frequency* before and after treatment was categorized based on number of episodes per year, into four different categories being ‘No UTI episodes’, ‘1‐6 UTI episodes per year’, ‘7 to 12 UTI episodes per year’ and ‘>12 UTI episodes per year’.^13^

*Multidrug resistance (MDR)* was defined as culture‐proven resistance to 3 or more antimicrobial classes.^21^

### 2.5 Study outcomes

Posttreatment patient‐reported outcome measures including Patient Global Impression of Improvement (PGI‐I) in symptoms, posttreatment QoL (also using the IPSS‐QoL) and presence of *treatment success* were recorded by following up the patients after their management.

The primary endpoint was treatment success at latest follow‐up. Treatment success was operationally defined as reduced frequency of UTI episodes by ≥50% or symptom improvement by ≥50% compared with baseline at latest follow‐up.

A UTI episode was defined as symptomatic recurrence requiring antibiotic treatment, with positive urine culture, where available. Symptom‐defined episodes were included where culture was unavailable.^13^

PGI‐I and post‐treatment QoL reported by patients are co‐primary outcomes.

### 2.6 Statistical analysis

Statistical analyses were conducted using SPSS software version 26.0. Descriptive statistics were utilised to summarize patient demographics and baseline disease characteristics, with categorical variables presented as frequencies and percentages, and continuous variables as mean, median, range and standard deviation. Bivariate analysis of categorical variables was performed using Pearson's chi‐square test, while independent sample *t* tests were applied to continuous variables. Additionally, multivariate analysis with binary logistic regression was employed to minimize confounding bias and establish statistical significance.

## 3 RESULTS

### 3.1 Baseline patient characteristics and investigations

Data were collected and analysed for 211 consecutive patients referred to our dedicated complex‐UTI clinic, who fulfilled the inclusion criteria during the study period. There was no missing or loss to follow up data to report, due to which no patients were excluded. Of the included 211, 107 (51%) patients were referred from the community by the GPs, whereas 104 (49%) patients were referred from specialist clinics. The baseline patient characteristics are as depicted in Table 1. The age of the patients ranged from 18 to 89 years, with mean age being 58.3 years (median = 60, standard deviation = 18.9). Of the 211 patients, 22 (10.4%) were male and 189 (89.6%) female. Postmenopausal women contributed to 65.4% (*n* = 138) of our patient population; 145 (69%) patients had significant comorbidities, with 59 (28%) patients being neuropathic and 24 (11.4%) being immunosuppressed. All the patients fit into the European Association of Urology (EAU) definition of recurrent UTIs, with 52 (24.6%) presenting with more than six episodes of UTI in a year; 24 (11.4%) patients had a history of at least one hospital admission due to urosepsis. Only 28 (13.3%) patients had history of first‐ and/or second‐line treatment being tried before referral to the complex‐UTI clinic. In nearly 87% (*n* = 183) patients, even first‐ and/or second‐line treatment options were not employed prior to the clinic referral.

**TABLE 1:** Baseline population characterstics and investigations.

Total number of patients included (*N*)	211
**Age**	
Age range	18–89 years
Mean	58.3 years
Median	60 years
Standard deviation	18.9
**Gender**	***n* (%)**
Male	22 (10.4%)
Female*	189 (89.6%)
*Postmenopausal status	138 (65.4%)
**Source of referral**	***n* (%)**
General Practitioner	107 (50.7%)
Specialist	104 (49.3%)
**Presence of significant comorbidities**	***n* (%)**
Yes	145 (68.7%)
No	66 (31.3%)
**Presence of neuropathy**	***n* (%)**
Yes	59 (28%)
No	152 (72%)
**Presence of immunosuppression**	***n* (%)**
Yes	24(11.4%)
No	187 (88.6%)
**Number of UTI episodes per year (pre‐treatment)**	***n* (%)**
Up to 6 per year	159 (75.4%)
7 to 12 per year	50 (23.7%)
>12 per year	2 (0.9%)
**Hospital admissions with urosepsis**	***n* (%)**
Yes	24 (11.4%)
No	187 (88.6%)
**Preclinic treatment**	***n* (%)**
1st and/or 2nd line treatment tried and failed	28 (13.3%)
1st and/or 2nd line treatment not tried	183 (86.7%)
**Bacterial growth on urine culture (>10** ^**5**^ **CFU/mL)**	***n* (%)**
Single organism: *E. coli*	114 (54%)
Single organism: Non‐*E. coli*	48 (22.7%)
Multiple Organisms	49 (23.3%)
**Multidrug resistance on urine culture sensitivities**	***n* (%)**
Yes	57 (27%)
No	154 (73%)
**Imaging**	***n* (%)**
Normal	119 (56.4%)
Abnormal^a^	41 (19.4%)
Not done	51 (24.2%)
**Flexible cystoscopy**	***n* (%)**
Normal	105 (49.8%)
Abnormal^b^	39 (18.4%)
Not done	67 (31.8%)

^a^ Simple renal cysts (*n* = 19), renal stones (*n* = 15), focal renal scarring (*n* = 4), angiomyolipoma (*n* = 2), renal mass (*n* = 1).

^b^ Cystitis cystica (*n* = 26), urethral stenosis (*n* = 9), bladder cancer (*n* = 2), postradiation changes (*n* = 2).

*Escherichia coli* was the most common organism grown on urine cultures and was the solitary causative organism in 114 (54%) patients. Multiple bacterial organisms were grown in 23% of the patients. MDR was documented in 27% of cases at presentation to the clinic.

Imaging in the form of ultrasonography of the renal tract was warranted for 160 (75.8%) patients. If ultrasound findings of renal stones, complex renal cysts or suspected renal mass were present, a further assessment with computerised tomography was performed.

The imaging did not reveal any abnormality in 119 cases, whereas the remaining 41 (19.4%) revealed renal findings of simple renal cysts (*n* = 19), renal stones (*n* = 15), focal renal scarring (*n* = 4), angiomyolipoma (*n* = 2) and renal mass (*n* = 1). Flexible cystoscopy was indicated and done for 144 (68.3%) patients and was normal in 105 patients, whereas in 18.4% (*n* = 39), cystoscopy revealed abnormalities including cystitis cystica changes in 26 patients, urethral stenosis in nine, postradiotherapy changes in two, with only two patients being diagnosed with bladder tumour.

At the time of initial presentation to the clinic, 141 (66.8%) and 70 (33.2%) patients reported pretreatment QoL as ‘poor’ and ‘satisfactory’, respectively, while none of the patients reported ‘good to excellent’ QoL (Table 2).

**TABLE 2:** Bivariate analysis of patient/disease factors with treatment success.

Patient/disease factor	Category (if applicable)	Treatment success		Odd's ratio (95% confidence interval)	Wald *p* value*	Overall *p* value** (*χ* ^2^/Fisher)
		Yes	No			
Age	‐	185 (87.7%)	26(12.3%)	‐		0.160
Gender	Female (reference)	168 (79.6%)	21 (9.9%)	1	‐	0.110
	Male	17 (8.1%)	5 (2.4%)	0.47 (0.17–1.29)	0.144	
Neuropathy	No (reference)	133 (63%)	19 (9%)	1	‐	0.550
	Yes	52 (24.7%)	7 (3.3%)	1.04 (0.47–2.31)	0.923	
Postmenopausal status	No (reference)	43 (20.4%)	8 (3.8%)	1	‐	0.090
	Yes	125 (59.2%)	13 (6.2%)	1.37 (0.76–2.44)	0.290	
	Not applicable	17 (8%)	5 (2.4%)	0.47 (0.17–1.29)	0.144	
Immunosuppressed state	No (reference)	169 (80.1%)	18 (8.5%)	1	‐	**<0.001**
	Yes	16 (7.6%)	8 (3.8%)	0.28 (0.12–0.65)	0.003	
Significant comorbidities	No (reference)	63 (29.9%)	3 (1.4%)	1	‐	**0.014**
	Yes	122 (57.8%)	23 (10.9%)	0.74 (0.47–1.16)	0.182	
UTI frequency per year (pre‐treatment)	≤6 episodes/year (reference)	136 (64.5%)	23 (10.9%)	1	‐	0.299
	7–12 episodes/year	47 (22.3%)	3 (1.4%)	2.22 (0.69–7.16)	0.184	
	>12 episodes/year	2 (0.9%)	0 (0%)	‐	‐	
Hospital admissions with urosepsis	No (reference)	170 (80.6%)	17 (8%)	1	‐	**0.001**
	Yes	15 (7.1%)	9 (4.3%)	0.23 (0.10–0.53)	<0.001	
Bacterial growth	Single organism: *E. coli* (reference)	103 (48.8%)	11 (5.2%)	1	‐	**0.047**
	Single organism: Non‐*E. coli*	44 (20.9%)	4 (1.9%)	1.55 (0.56–4.35)	0.398	
	Multiple organisms	38 (18%)	11 (5.2%)	0.48 (0.24–0.94)	0.035	
Pretreatment multidrug resistance	No (reference)	140 (66.4%)	14 (6.6%)	1	‐	**0.020**
	Yes	45 (21.3%)	12 (5.7%)	0.52 (0.27–0.99)	0.049	
Imaging	Normal (reference)	104 (49.3%)	15 (7.1%)	1	‐	0.900
	Abnormal	36 (17.1%)	5 (2.4%)	1.01 (0.40–2.59)	0.983	
	Not done	45 (21.3%)	6 (2.8%)	1.05 (0.45–2.49)	0.905	
Flexible cystoscopy	Normal (reference)	95 (45.2%)	10 (4.7%)	1	‐	**0.020**
	Abnormal	29 (13.7%)	10 (4.7%)	0.40 (0.19–0.83)	0.015	
	Not done	61 (28.9%)	6 (2.8%)	1.44 (0.62–3.34)	0.398	
Preclinic treatment	1st and/or 2nd line treatment not tried (reference)	162 (76.8%)	21 (9.9%)	1	‐	0.248
	1st and/or 2nd line treatment tried and failed	23 (10.9%)	5 (2.4%)	0.64 (0.24–1.70)	0.370	
Pretreatment quality of life	Poor QoL (reference)	119 (56.4%)	22 (10.4%)	1	‐	**0.040**
	Satisfactory QoL	66 (31.3%)	4 (1.9%)	2.35 (0.85–6.47)	0.099	
	Good to excellent QoL	0 (0%)	0 (0%)	‐	‐	
Postinvestigation intervention	No (reference)	168 (79.7%)	22 (10.4%)	1	‐	0.482
	Yes	17 (8%)	4 (1.9%)	0.60 (0.20–1.78)	0.358	
Tertiary treatment	No tertiary treatment (reference)	141 (66.8%)	9 (4.3%)	1	‐	**<0.001**
	Intravesical antibiotics	24 (11.4%)	12 (5.7%)	0.28 (0.14–0.56)	<0.001	
	Intravesical GAG	20 (9.4%)	5 (2.4%)	0.56 (0.21–1.50)	0.250	

* Odds ratios (ORs) with 95% confidence intervals (CIs) are crude (unadjusted) effect estimates comparing each category with the stated reference category. Category‐specific *p* values are two‐sided Wald *p* values derived from the reported ORs and 95% CIs.

** Overall *p* values were calculated using Pearson's *χ* ^2^ test or Fisher's exact test where appropriate.

### 3.2 Treatment received and patient reported outcome measures

In the clinic, all 211 (100%) patients were treated using first‐ and/or second‐line treatment modalities. The options of antibiotic and nonantibiotic therapies were discussed, and patients were involved, allowing for shared decision making. In addition, tertiary treatment in the form of intravesical therapies was required for 61 (29%) patients. Of these patients, 36 patients were managed with intravesical antibiotic therapy, whereas 25 patients were managed with intravesical glycosaminoglycan therapy.

Surgical interventions following investigative findings from cystoscopy or imaging were performed in 21 patients. These included procedures for obstructive renal stones in nine patients, urethral stenosis in nine, bladder cancer in two and a renal mass in one patient.

**FIGURE 2:**
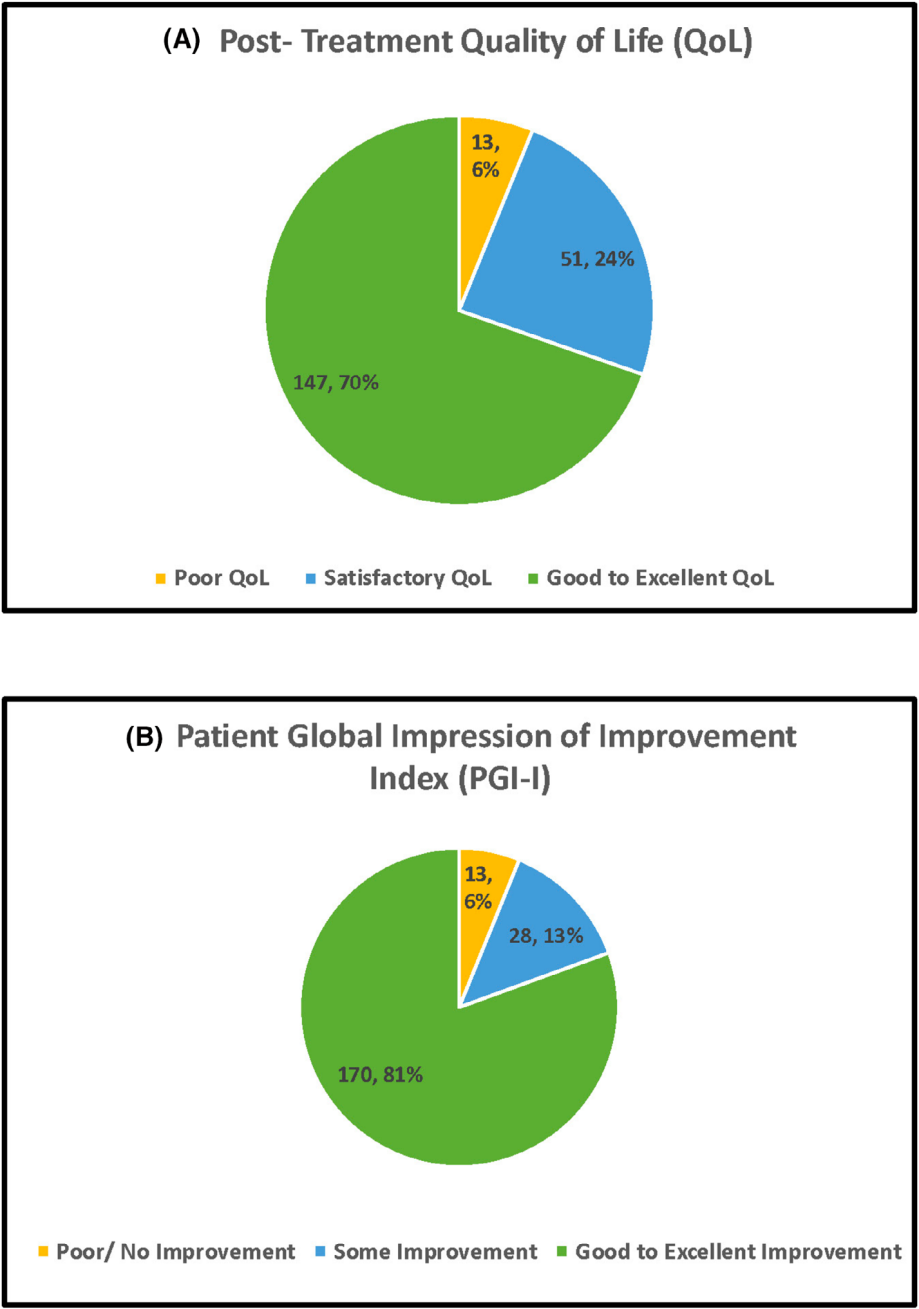
Patient reported outcome measures. (A) Posttreatment quality of life (Qol). (B) Patient Global Impression of Improvement Index (PGI‐I).

**FIGURE 3:**
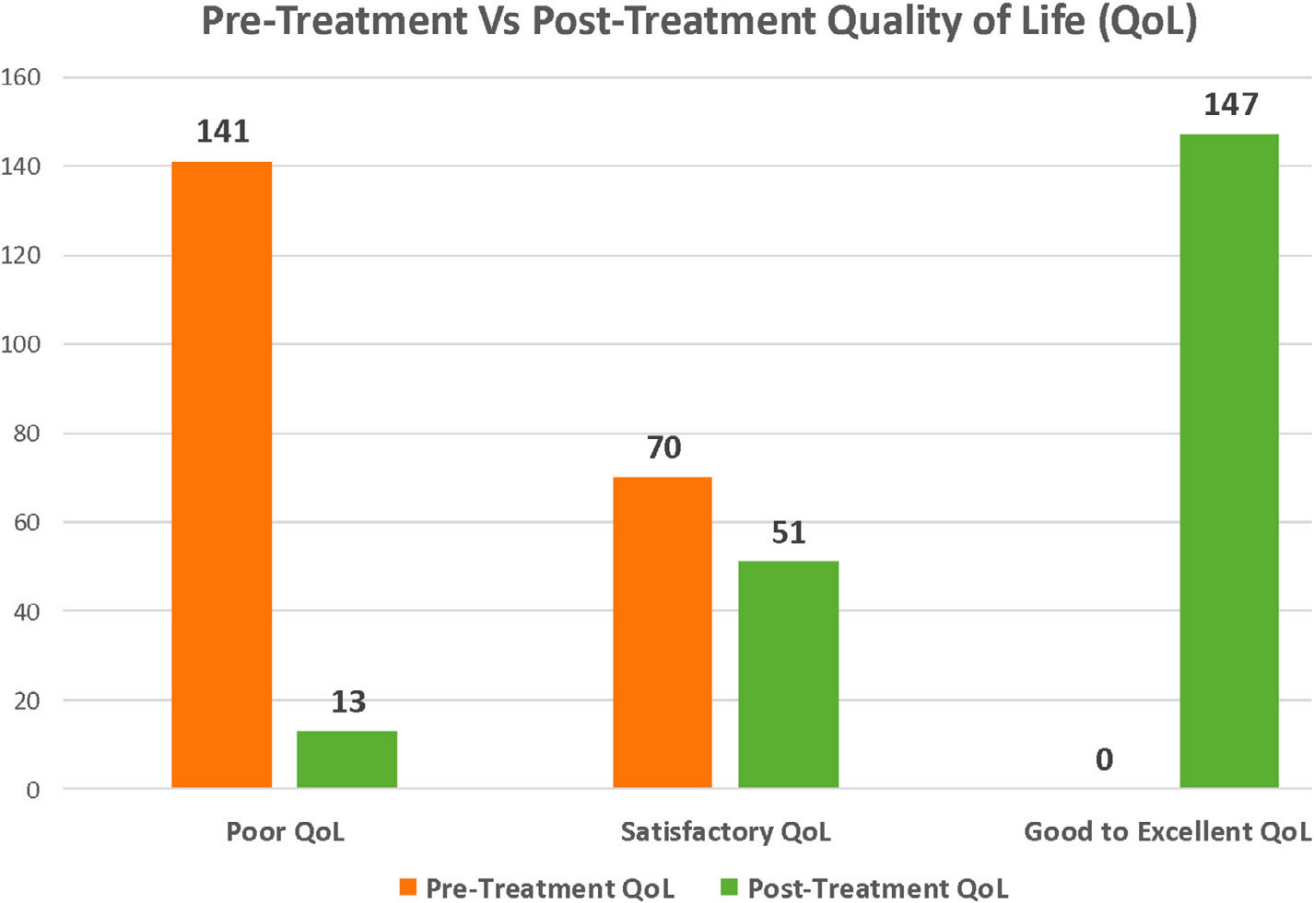
Pretreatment versus posttreatment quality of life (Qol).

**FIGURE 4:**
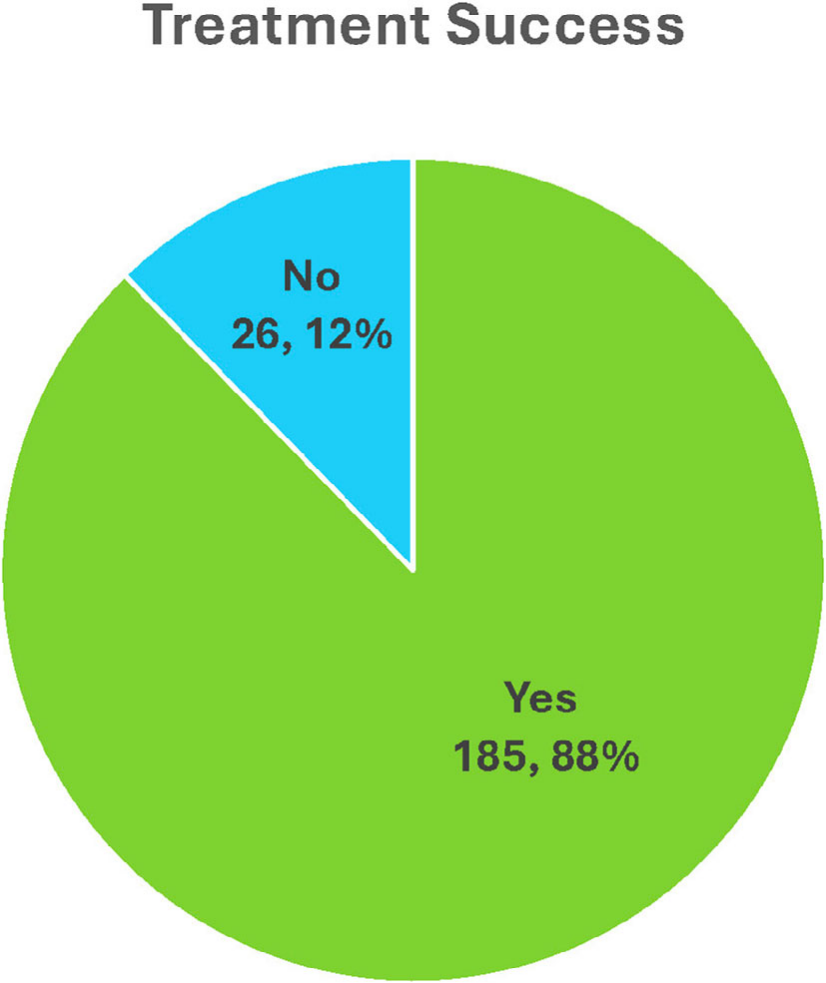
Treatment success.

All the 211 patients were followed up for a minimum of 6 months after being on the devised treatment plan. The median follow up period was 16 **±** 7.1 months. On assessing the patients' impression of symptomatic improvement using the PGI‐I scale, around 81% (*n* = 170) and 13% (*n* = 28) patients reported good to excellent and some improvement respectively, whereas 6% (*n* = 13) patients reported poor or no improvement. Post‐Treatment QoL assessment revealed 70% patients reporting good to excellent QoL post‐treatment (Figure 2). The QoL assessment demonstrated a significant improvement post‐treatment, depicted by the right shift across the three QoL categories (Figure 3). Overall treatment success, as per the pre‐established definition was documented in 87.7% (*n* = 185) patients (Figure 4). The included cohort had no patients that were lost to follow‐up and there were no refusals or missing data to report.

### 3.3 Bivariate analysis

A bivariate analysis was used to identify several significant patient and disease factors associated with treatment success (Table 2). The presence of significant comorbidities was found to have a notable association (*p* value = 0.014). Immunosuppressed state also showed a significant association with the outcome of treatment success, with a *p* value of <0.001. The presence of a history of hospital admission for urosepsis also showed significance with a *p* value of 0.001. Bacterial growth on urine cultures (*p* value = 0.047), pretreatment multidrug resistance pattern (*p* value <0.020), presence of abnormalities on flexible cystoscopy (*p* value = 0.020), pretreatment QoL (*p* value = 0.040) and receiving tertiary treatment (*p* value <0.001) showed statistical significance (*p* value <0.05) in being associated with treatment success.

However, other variables did not show significant associations with treatment success. Age, gender, presence of neuropathy or postmenopausal status did not significantly impact the outcomes. There was no significant difference in success rates between the male and female patients (*p* value = 0.110). Presence of Neuropathy (*p* value = 0.550) and postmenopausal status (*p* value = 0.090) did not reach significance to be associated with treatment success and there was no significant difference across UTI frequency categories(*p* value = 0.299). Similarly, the presence of abnormality on imaging (*p* value = 0.900) and needing postinvestigation surgical intervention (*p* value = 0.482) was not significantly associated with treatment success.

### 3.4 Multivariate analysis

The multivariate analysis, performed as an extension, confirmed the significance of few variables in predicting treatment success while adjusting for potential confounding bias. Four variables are statistically significant in the multivariate regression analysis (Table 3). These included immunosuppressed state (*p* value = 0.012), presence of significant comorbidities (*p* value = 0.004), history of hospital admission for urosepsis (*p* value <0.001) and requirement of tertiary treatment (*p* value <0.001), which were proven to be significant independent factors for predicting treatment success.

**TABLE 3:** Multivariate analysis of patient/disease factors with treatment success.

Patient/disease factor	*p* value on bivariate analysis	Corrected *p* value on multivariate analysis
Immunosuppressed state	<0.001	0.012
Significant comorbidities	0.014	0.004
Hospital admissions with urosepsis	0.001	<0.001
Bacterial growth	0.047	0.132
Pretreatment multidrug resistance	0.020	0.118
Flexible cystoscopy	0.020	0.311
Pretreatment quality of life	0.040	0.200
Tertiary treatment	<0.001	<0.001

## 4 DISCUSSION

Effective management of chronic clinical conditions, including recurrent urinary tract infections (rUTIs), begins with fundamental principles of care: a comprehensive assessment, addressing modifiable risk factors and leveraging input from multidisciplinary teams to ensure holistic treatment. Our dedicated UTI clinic exemplifies this approach by integrating these essential elements. One notable example is the frequent use of nitrofurantoin as a primary treatment for UTIs. When treatment is ineffective, clinicians must consider factors such as reduced renal function, which diminishes nitrofurantoin's efficacy, and exercise caution when prescribing it to older adults or patients with pre‐existing pulmonary conditions.^22^ Dedicated UTI clinics are uniquely positioned to address such specific concerns through the expertise of microbiologists and multidisciplinary discussions, ensuring tailored and effective care for each patient.

Over the past decade, dedicated UTI clinics have been established globally, guided by a multidisciplinary framework outlined in limited existing literature.^23^ However, our study is the first‐of‐its‐kind to report treatment outcomes from a specialized multidisciplinary UTI clinic. Our results highlight the contribution of every member of the team, with their specialist input such as the functional urology specialist nurses supporting the clinical care such as encouraging and tutoring for intravesical self‐instillations. They also empower the patients to continuing their treatment in a supportive environment with close face‐to‐face and telephone follow‐up.

A key finding of our study is that 71% of patients showed a positive response to first‐ and second‐line treatments, which included several non‐antibiotic options such as methenamine hippurate, probiotics and hormonal supplementation. These treatments were administered either independently or in combination with low‐dose prophylactic antibiotics. Our results are consistent with evidence from the previous studies that have highlighted the efficacy of these interventions. For instance, a multicentre, open‐label randomized controlled trial by Harding et al. found methenamine hippurate to be noninferior to daily low‐dose antibiotics in preventing rUTIs in women.^11^ Similarly, a double‐blind, placebo‐controlled study by Gupta et al. demonstrated the effectiveness of both vaginal and oral probiotics in reducing symptomatic rUTI episodes.^24^ Additionally, the topical application of vaginal oestrogen has gained attention due to its proven ability to prevent rUTI episodes while maintaining a low systemic side effect profile.^25^, ^26^, ^27^

Though our study demonstrates the success of noninvasive treatment modalities, as reflected in our results, it also highlights a low adherence rate (approximately 13%) among primary and secondary care clinicians to prescribe first‐ and second‐line treatments before referring to our clinic. This underscores the need to raise awareness across all healthcare settings about the efficacy, safety and simplicity of these treatment options. Increasing awareness could facilitate earlier intervention and reduce the burden on an overbooked clinic, minimizing delays in patient care.

When the dedicated UTI clinic was established, it was designed to support patients perceived as having ‘complex rUTI’, where *complexity was operationally defined* as persistence and repeated recurrence despite guideline‐based treatment, risk of multidrug‐resistant organisms, significant comorbidity burden and/or diagnostic uncertainty. However, as mentioned, a substantial 71% of referred patients improved with first‐ and second‐line noninvasive interventions alone highlighting that not all referrals represented inherently complex cases. This is a key service outcome of our study, and it suggests that, with robust implementation of guideline‐based pathways (AoMRC and EAU), many patients may be effectively managed in primary care, with tertiary referral focused on those with refractory disease or higher risk features.^14^, ^15^

Recent updates to NICE guidance (December 2024) place greater emphasis on structured assessment, self‐care strategies and evidence‐based prophylaxis options for recurrent UTI, supporting expanded management within primary care.^28^ This is in line with our findings, suggesting a shift in emphasis to the opportunity for pathway redesign, reducing unnecessary tertiary referrals and prioritizing specialist clinic capacity for those requiring third‐line interventions.

In our cohort, 29% of patients required escalation to third line/tertiary interventions after inadequate response to conservative and second‐line strategies. This group included patients managed with intravesical antibiotic therapy or intravesical glycosaminoglycan therapy, reflecting refractory symptoms, recurrence despite standard prophylaxis and/or limitations posed by antimicrobial resistance or intolerance to systemic agents. The multidisciplinary model is particularly advantageous in these cases, as microbiology input supports targeted therapy and stewardship, while specialist nurse support enables patient education and safe intravesical self‐instillation where appropriate. Importantly, the requirement for tertiary therapy was significantly associated with poorer overall response on regression analyses, highlighting that this subgroup may represent a genuinely complex rUTI phenotype requiring specialist input.^13^

Additionally, our findings revealed a low percentage (fewer than 20%) of patients exhibiting abnormalities among those who underwent flexible cystoscopy, with even fewer requiring surgical intervention or warranting treatment modification, like those with urethral stenosis and bladder cancer. This highlights the limited role of flexible cystoscopy as a routine investigation in rUTI patient cohorts. Hence, consistent with existing literature, we recommend selective use of cystoscopy, focusing on patients with red‐flag symptoms such as haematuria or nonresponsiveness to all noninvasive treatments, to enhance its diagnostic yield.^15^, ^29^, ^30^, ^31^

Our results demonstrate that rUTIs significantly affect QoL. Accordingly, our study evaluated treatment outcomes using patient‐reported measures such as PGI‐I scores and posttreatment QoL assessments, alongside overall treatment success, emphasizing a patient‐centred clinical approach. It is also crucial to manage patient expectations early, particularly for those with irreversible risk factors, by emphasizing improvements in QoL and reducing the frequency and severity of symptoms rather than aiming for complete resolution of UTIs.

The chronic persistent nature of rUTIs, complicated with debilitating symptoms such as pain, urgency and frequency, also disrupts daily activities, sleep and social interactions, leading to emotional distress. While our clinic ensures regular patient correspondence and support through functional urology specialist nurses, we recognize the existing gap in providing comprehensive mental health services. To address this, we plan to integrate in‐house psychological support by involving clinical psychologists, aiming to offer holistic care as a future initiative.

As the first study in this area, its added strength lies in the inclusion of a large patient cohort with adequate follow‐up periods, enabling meaningful conclusions to be drawn regarding various aspects of patient management.

The authors have also noted that no data were missing, and no patients were lost to follow‐up, despite the inclusion of 211 patients with substantial follow‐up durations. This was achieved through a clinic structure that supported robust follow‐up at regular intervals, largely facilitated by functional urology specialist nurses. Follow‐up was frequently conducted via telephone follow‐up consultations when face‐to‐face review was not feasible due to patient‐related factors. Patients were also provided with direct contact details to report any treatment‐ or symptom‐related concerns requiring medical attention. This underscores the importance of the utility of the wider team.

This study has certain constraints, including the absence of a control arm, which limits direct comparisons. QoL outcomes were derived using the QoL component of the IPSS Score, chosen for its simplicity and ease of use in a busy clinical setting. Although a validated UTI questionnaire format^32^ was used in the clinic, its QoL responses were not included in the data analysis due to incomplete patient responses in this section. To address this, we plan to implement an electronic questionnaire system in the future to ensure all fields are completed to enable submission, enhancing data accuracy and consistency.^33^

In addition, although we used the IPSS QoL component due to its simplicity and feasibility, future work would benefit from disease‐specific validated Recurrent UTI Impact Questionnaire (RUTIIQ), which may better capture symptom burden, psychosocial impact and treatment‐related change.^34^

The authors suggest that the outcomes achieved in this dedicated clinic can be effectively applied to primary and secondary care settings by ensuring that patients receive appropriate and timely first‐ and second‐line treatments prior to referral to a specialist clinic. This strategy has the potential to optimize the overbooked clinic's workload and facilitate the prompt evaluation of patients who are most likely to benefit from the multidisciplinary input, thereby enhancing clinic efficiency and improving patient outcomes.

A comparative analysis of baseline characteristics and treatment outcomes between patients who required formal MDT input and those who did not was beyond the scope of the current study. However, we recognize this as an important area for further investigation and intend to explore it in detail in a future, dedicated analysis.

In conclusion, dedicated UTI clinics play a crucial role in improving treatment outcomes of rUTIs and enhancing patients' QoL through the application of evidence‐based interventions and collaborative, multidisciplinary expertise that adds a broadened clinical perspective. To further optimize patient outcomes, it is essential to promote awareness of effective noninvasive treatment options throughout the healthcare system and implement a selective approach to diagnostic procedures, fostering efficiency and prioritizing patient‐centred care.

## AUTHOR CONTRIBUTIONS

**Pragnitha Chitteti:** Data curation; formal analysis; investigation; methodology; writing—original draft; writing—review and editing; visualisation. **Ekpeno Inyang:** Data curation; investigation; writing—review and editing. **Ahmed Ghonaimy:** Data curation; investigation; writing—review and editing. **Jayne Morris‐Laverick:** Data curation; investigation; writing—review and editing. **Stephanie Bezemer:** Data curation; investigation; writing—review and editing. **Igor Kubelka:** Writing—review and editing. **Victoria McCune:** Writing—review and editing. **Mehwash Nadeem:** Conceptualisation; methodology; formal analysis; supervision; project administration; writing—review and editing; final approval of the manuscript.

## CONFLICT OF INTEREST STATEMENT

The authors declare no conflict of interest.

## Supporting information


**Data S1.** Supporting Information
