# Supplementary material for: Treatment outcomes of multidisciplinary management of recurrent urinary tract infections: A 2‐year experience in a dedicated complex‐UTI clinic
Source: BJUI Compass. 2026 Mar 4;7(3):e70177. doi: 10.1002/bco2.70177 (PMC12960744; doi:10.1002/bco2.70177)
Supplement: Supplementary file 1 — Data S1. Supporting Information [file BCO2-7-e70177-s001.pdf]

South Tees Hospitals
NHS Foundation Trust

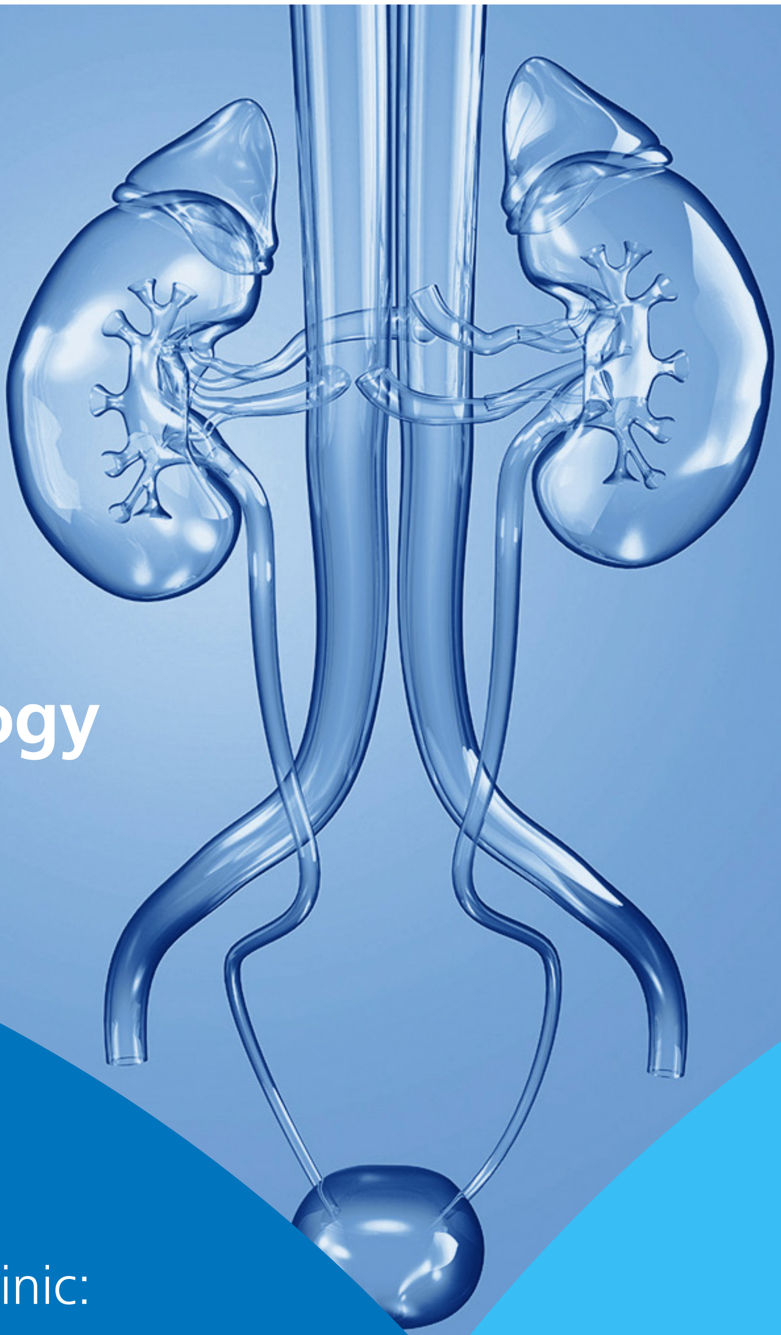

Functional, Reconstructive and Neuro-Urology

Recurrent and Complex Urinary Tract Infection Clinic:

Information Booklet and Questionnaire

Department of Urology-
UTI Service

Why you are referred to the Recurrent/Complex UTI clinic?

You have been referred to this specialist clinic by your GP/ consultant because of the urinary infections that you have had.

In this clinic, we aim to:

1. Review your symptoms and provide thorough consultation
2. Ensure that you have been offered all relevant investigations
3. Discuss all possible treatment approaches with you in detail

A Consultant Urologist Ms Mehwash Nadeem and Consultant Microbiologist Dr Victoria McCune and Dr Igor Kubelka alongside our specialist nurses, Sr Jayne Morris Laverick and Sr Stephanie Bezemar, will all input into the advice and management strategy you receive.

You can expect to attend this clinic 2- 3 times, on the first visit you may be asked submit a urine sample and you may also have some blood tests. It is important that this urine sample is collected as cleanly as possible and instructions on how to do this will be given to you on arrival at the clinic.

If you are taking antibiotics on a daily basis for your urinary problems, we would like you to stop these 5 days before the clinic if possible. This makes it more likely that we will be able to detect bacteria in your urine.

We would like you to fill out the attached questionnaire as much as possible and kindly bring it with you to the clinic. This will help us to understand the issue from your perspective. Our team will endeavour to help and support you throughout your treatment.

Patient details

Name:

Date of Birth:

Hospital Number:

NHS Number:

Referrer

GP/Consultant Name:

Address: ....

....

....

Symptoms

1. When did you first start getting urine infections?

- ☐ Less than six months ago
- ☐ 6-12 months ago
- ☐ 1 - 2 years ago
- ☐ 2 - 5 years ago
- ☐ More than 5 years ago
- ☐ Since childhood (age of first infection ....)

2. How many infections have you had in the last:

- ☐ 6 months ....
- ☐ 12 months ....

3. Is there anything in particular 'triggers' your urinary infection (sexual intercourse, constipation etc)?
If so, please describe here

4. What symptoms do you get with a urine infection? (Tick all that apply)

- ☐ Burning and/or stinging when passing urine
- ☐ Burning and/or stinging after passing urine
- ☐ Passing urine frequently
- ☐ Rushing to the toilet
- ☐ New onset of nocturia
- ☐ Pains in tummy
- ☐ Pains in sides or back
- ☐ Fever
- ☐ Not listed above (please describe below)

5. In a month – approximately how many days will you have symptoms?

....

6. On a scale of 1-10 where 10 is the worst infection you have had how bad are your symptoms this week?

....

Please complete attached questionnaire for quality-of-life assessment
(UTISA questionnaire - to be attached)

7. Have you been admitted to hospital with these infections? When was the last time?

....

....

....

Previous Surgical history

8. Have you had any urological surgery in past? (please specify in as much details as possible)

9. Have you had any previous bowel surgery? Details?

....

....

....

Previous gynaecological history

(Male patients to skip to next section please)

10. Have you had any previous gynaecological surgery? Details?

....

....

11. Have you had children? How many? Any complications around their delivery?

....

....

12. Do you suffer with prolapse in any part of your pelvic floor?

....

....

13. Are you still having regular periods or have you gone through the menopause?

....

....

Other medical problems

14. Please list any other medical problems you have

....

....

....

....

....

....

Treatments

15. Which antibiotics have you tried in the past?

16. Are you allergic to any antibiotics? Please give details, please tell us what happens when you take the antibiotic and how long ago the reaction was

17. Have you tried any of the below ways to prevent urine infections? (Please tick)

- ☐ Cranberry capsules
- ☐ Methenamine hippurate/ Hiprex
- ☐ D-Mannose
- ☐ Oestrogen creams
- ☐ Other – please give details

To ensure we meet your communication needs please inform the Patient Experience Department of any special requirements, i.e. Braille/ Large Print.

T: 01642 835964
E: stees.patient.experience@nhs.net

The James Cook University Hospital,
Marton Road, Middlesbrough, TS4 3BW. Tel: 01642 850850
Issue Date: February 2021 Review Date: February 2023

ST1677
